# Comparison of systemic inflammatory profiles in COVID-19 and community-acquired pneumonia patients: a prospective cohort study

**DOI:** 10.1186/s12931-023-02352-2

**Published:** 2023-02-22

**Authors:** Elsa D. Ibáñez-Prada, Matthew Fish, Yuli V. Fuentes, Ingrid G. Bustos, Cristian C. Serrano-Mayorga, Julian Lozada, Jennifer Rynne, Aislinn Jennings, Ana M. Crispin, Ana Maria Santos, John Londoño, Manu Shankar-Hari, Luis Felipe Reyes

**Affiliations:** 1 Universidad de La Sabana, Campus Puente del Común, KM 7.5 Autopista Norte de Bogotá, Chia, Colombia; 2 Centre for Inflammation Research, University of Edinburgh, 47 Little France Crescent, Edinburgh, Scotland, UK; 3 Clínica Universidad de La Sabana, Chía, Colombia; 4 Pandemic Sciences Institute, University of Oxford, Oxford, UK

**Keywords:** COVID-19, Community-acquired pneumonia, Cytokines, Immunology

## Abstract

**Background:**

Inflammatory responses contribute to tissue damage in COVID-19 and community-acquired pneumonia (CAP). Although predictive values of different inflammatory biomarkers have been reported in both, similarities and differences of inflammatory profiles between these conditions remain uncertain. Therefore, we aimed to determine the similarities and differences of the inflammatory profiles between COVID-19 and CAP, and their association with clinical outcomes.

**Methods:**

We report a prospective observational cohort study; conducted in a reference hospital in Latin America. Patients with confirmed COVID-19 pneumonia and CAP were included. Multiplex (Luminex) cytokine assays were used to measure the plasma concentration of 14 cytokines at hospital admission. After comparing similarities and differences in the inflammatory profile between COVID-19 and CAP patients, an unsupervised classification method (i.e., hierarchical clustering) was used to identify subpopulations within COVID-19 and CAP patients.

**Results:**

A total of 160 patients were included, 62.5% were diagnosed with COVID-19 (100/160), and 37.5% with CAP (60/160). Using the hierarchical clustering, COVID-19 and CAP patients were divided based on its inflammatory profile: pauci, moderate, and hyper-inflammatory immune response. COVID-19 hyper-inflammatory subpopulation had the highest mortality. COVID-19 hyper-inflammatory subpopulation, compared to pauci-inflammatory, had higher levels of IL-10 (median [IQR] 61.4 [42.0–109.4] vs 13.0 [5.0–24.9], *P*: < 0.001), IL-6 (48.1 [22.3–82.6] vs 9.1 [0.1–30.4], *P*: < 0.001), among others. Hyper-inflammatory vs pauci-inflammatory CAP patients were characterized by elevation of IFN2 (48.8 [29.7–110.5] vs 3.0 [1.7–10.3], *P*: < 0.001), TNFα (36.3 [24.8–53.4] vs 13.1 [11.3–16.9], *P*: < 0.001), among others. Hyper-inflammatory subpopulations in COVID-19 and CAP compared to the corresponding pauci-inflammatory subpopulations had higher MCP-1.

**Conclusions:**

Our data highlights three distinct subpopulations in COVID-19 and CAP, with differences in inflammatory marker profiles and risks of adverse clinical outcomes.

*Trial registration:* This is a prospective study, therefore no health care intervention were applied to participants and trial registration is not applicable.

**Supplementary Information:**

The online version contains supplementary material available at 10.1186/s12931-023-02352-2.

## Introduction

Severe acute respiratory syndrome coronavirus 2 (SARS-CoV2) is a highly transmissible pathogen that emerged in 2019, causing Coronavirus disease 2019 (COVID-19) [1]. Since the onset of the COVID-19 pandemic, up to 700 million cases have been documented, including around 7 million deaths to date [2]. COVID-19 is a multi-systemic disease [3], with respiratory failure secondary to pneumonitis being the most common presentation of severe infection [4]. COVID-19 pneumonia is associated with elevated inflammatory cytokines and dysregulated inflammation-causing lung and other end-organ damage [5, 6].

Before the COVID-19 pandemic, lower respiratory tract infections (LRTI) were the leading cause of mortality and morbidity worldwide, representing the fourth cause of mortality for all ages in 2020 [5, 6]. The LRTI case fatality rate is around 23% in patients admitted to the intensive care units (ICU) [7]. Clinically, COVID-19 pneumonitis and community-acquired pneumonia (CAP) are community-acquired LRTIs [8]. Biologically, both COVID-19 pneumonitis and CAP are associated with dysregulated systemic inflammation. Inflammation in COVID-19 patients is associated with up-regulation of interleukin (IL)-2, IL-6, IL-10, granulocyte colony-stimulating factor (G-CSF), interferon-inducible protein 10 (IP-10), monocyte chemoattractant protein 1 (MCP1) [9]. In sharp contrast, inflammation in CAP patients is characterized by elevation of the cytokines such as interleukin (IL)-4, IL-6, IL-10, IL-8, IL-1β, tumour necrosis factor (TNF)-α, and other factors which belongs to the T-helper (Th) 17 subset [10]. However, few studies have compared this dysregulated inflammatory profile between COVID-19 and CAP. In this observational cohort study, we hypothesized that there would be similarities, but the differences in the inflammatory profile between COVID-19 and CAP will be associated with clinical outcomes.

## Materials and methods

This observational prospective cohort study of subjects admitted to the Clínica Universidad de La Sabana in Chía, Colombia, with LRTI. All consecutive patients admitted to the participating centre between November 2019, and May 2020 were included in the study. Data were collected prospectively by the attending physicians by reviewing medical records, laboratory data, and blood samples within the first 24 h of hospital admission were gathered to carry out the cytokine characterization. This study was approved by the Institutional Review Board (IRB) of the Clínica Universidad de La Sabana, and all patients signed informed consent to participate in the study.

### Subjects and data collection

The cohort includes hospitalized patients older than 18 years with an LRTI diagnosis based on the current American Thoracic Society and the Infectious Diseases Society of America (ATS/IDSA) guidelines [11] during the first 24 h of hospital admission. Those patients with documented co-infection at hospital/ICU admission were excluded. Pneumonia was defined as suggestive clinical features and a chest X-ray or other imaging assessment documenting alveolar infiltrates. Moreover, patients with at least three minor criteria or one major criterion of the ATS/IDSA severity criteria were diagnosed with severe CAP. Being minor criteria: respiratory rate ≥ 30 breaths/min, PaO_2_/FiO_2_ ratio ≤ 250, multilobar infiltrates, confusion/disorientation, uraemia (blood urea nitrogen level, 20 mg/dL), leukopenia (white blood cell count, < 4000 cells/mm^3^), thrombocytopenia (platelet count, < 100,000 cells/mm^3^), hypothermia (core temperature, 36ºC), hypotension requiring aggressive fluid resuscitation. The major criteria were invasive mechanical ventilation or septic shock with the need for vasopressors [11].

SARS-CoV-2 infection was documented by reverse transcription-polymerase chain reaction (rt-PCR) in a respiratory sample in a centralized laboratory. COVID-19 severity was also defined with ATS/IDSA COVID-19 guidelines [12]. Severe illness was diagnosed in patients with SpO^2^ ≤ 94% on room air, including patients on supplemental oxygen, oxygen through a high-flow device, or non-invasive ventilation. Critical illness was diagnosed in patients requiring invasive mechanical ventilation and/or extracorporeal membrane oxygenation (ECMO), or end-organ dysfunction [12].

During hospital admission, the following variables were gathered: demographic data, comorbidities, symptoms, physiological variables collected during the first 24 h of ICU admission, systemic complications, and laboratory reports. Additionally, a retrospective chart review was carried out at hospital discharge to double-check the registered data.

### Blood samples for cytokine analysis and cytokine identification

Venous blood was collected using EDTA tubes within the first 24 h of hospital admission. Blood samples were centrifuged at 1000 × *g* for 10 min within 30 min of blood collection. The plasma was removed and froze at − 80 °C in aliquots until cytokines analysis. The samples were thawed completely, mixed, and centrifuged before being used in the assay to remove particulates.

Multiplex (Luminex) cytokine assays were used to measure plasma cytokine concentrations. The assay was conducted using 25 μL of plasma sample, and cytokines were determined by standard curve analysis. The measured cytokines were basic fibroblast growth factor (FGF-2), Eotaxin, granulocyte–macrophage colony-stimulating factor (GM-CSF), interferon α-2 (IFNα-2), interferon-γ (IFN-γ), IL-10, IL-15, IL-1α, IL-6, IL-8, IP-10, monocyte chemoattractant protein-1 (MCP-1), macrophage inflammatory protein-1β (MIP-1β), and tumour necrosis factor-α (TNF-α). The Human Cytokine/Chemokine Magnetic Bead Panel kit from Millipore (HCYTOMAG-60 K) (Merck) was used to determine the cytokine plasma concentration. All reagents were provided with the kit and were prepared according to the manufacturer's recommendations.

Briefly, the antibody-bead vials were sonicated for 30 s and vortexed for 1 min. Then, 60 µL from each antibody bead vial was added to the mixing bottle, and 1.68 mL of bead diluents was added to bring the final volume to 3.0 mL. According to the manufacturer's instructions, the standards, quality controls, and serum matrix were reconstituted. The standards were serially diluted 1 to 5 [0 (Background), 3.2, 16, 80, 400, 2.000, and 10.000 pg/mL] in assay buffer as recommended by the manufacturer. Assay buffer was used for background wells. One quality control was also included in the study.

The 96-well plates were read and analysed on MAGPIX® System instrument using xPONENT® Software version 4.2. Standard curves were drawn for each cytokine. Then, cytokines concentrations were determined from the standard curve using a 5-point regression to transform the median fluorescence intensity values into concentrations for each analyte evaluated. Any value below the detection level was replaced by the limit of detection (LOD) as reported by the Luminex kit. Further information about the protocol has been reported previously elsewhere.

### Statistical analysis

All statistical analysis took place in RStudio. Cytokines included in the analysis were FGF-2, Eotaxin, GM-CSF, IFNα-2, IFN-γ, IL-10, IL-15, IL-1α, IL-6, IL-8, IP-10, MCP-1, MIP-1β, and TNF-α. Missing data for these cytokines were imputed using Gsimp package accounting for the lower limit of detection of the assay. Data was converted to a log2 scale to compare COVID-19 and CAP patients. We applied multiple comparisons in the limma package in a model with Benjamini Hochberg.

Data were visualized using Uniform Manifold Approximation and Projection for Dimension Reduction (UMAP), complex heatmap, and ggplot2. An unsupervised clustering analysis in the COVID-19 patients was performed to divide them according to their inflammatory profile: pauci-inflammatory immune response, moderate-inflammatory immune response, and hyper-inflammatory immune response. The data were log2 scaled and reduced to two dimensions using UMAP. COVID-19 patients were clustered based on UMAP variables. The number of subpopulations was determined by elbow, silhouette, and gap statistics in Nbclust and factoextra. Distance matrices and hierarchal clustering was done using Euclidean and ward.D method. Marker enrichment modelling (MEM) was used to identify discriminant cytokines of each group (see Additional file 1). COVID-19 patient subpopulations were compared to the CAP cohort in the limma package.

Regarding the clinical data, descriptive and bivariate analysis of the information was performed to determine the association between inflammatory profile and clinical outcomes, such as in-hospital mortality, the requirement of invasive mechanical ventilation, ICU admission, and hospital length of stay. Categorical variables are presented in counts (percentages) and were evaluated through the Chi-square test or Fisher's exact test. Continuous variables with normal distribution are expressed as means (standard deviation); variables with no normal distribution are expressed as median (interquartile ranges). For continuous variables with normal distribution, the t Student test was performed, and for variables with no normal distribution Wilcoxon-Mann–Whitney test was used.

## Results

The study cohort consisted of 160 hospitalized patients, 62.5% (100/160) COVID-19, and 37.5% (60/160) had CAP. Patients with COVID-19 were younger than those with CAP (57.0 [47.8–67.0] vs 64.0 [54.0–78.3]), and the majority were male (65.0% [65/100] vs 53.3% [32/60]). In the whole cohort, the most frequent comorbidities were arterial hypertension 38.1% (61/160), followed by chronic obstructive pulmonary disease (COPD) 11.3% (18/160), and diabetes mellitus 10.6% (17/160). A total of 70 patients (43.8%) patients admitted to the ICU, 49.0% (49/100) had COVID and 35.0% (21/60) CAP. Patients with COVID-19 had higher mortality when compared to those with CAP (17.0% [17/100] vs. 8.3% [5/60]), Table 1.

**Table 1:** Baseline characteristics of COVID-19 and CAP patients

Characteristic	All (N = 160)	COVID-19 patients (N = 100)	CAP patients (N = 60)	*P*-value
Male gender, N (%)	97 (60.6)	65 (65.0)	32 (53.3)	0.20
Age, median (IQR)	60.5 (48.0–71.3)	57.0 (47.8–67.0)	64.0 (54.0–78.3)	**0.01**
Comorbid conditions, N (%)				
Stroke	5 (3.1)	1 (1.0)	4 (6.7)	0.13
Myocardial infarction	3 (1.9)	0 (0.0)	3 (5.0)	0.10
Heart arrhythmia	4 (2.5)	2 (2.0)	2 (3.3)	1.00
Asthma	2 (1.3)	0 (0.0)	2 (3.3)	0.27
Bronchiectasis	1 (0.6)	0 (0.0)	1 (1.7)	0.80
Active cancer	4 (2.5)	1 (1.0)	3 (5.0)	0.30
Dementia	2 (1.3)	0 (0.0)	2 (3.3)	0.27
Diabetes mellitus	17 (10.6)	8 (8.0)	9 (15.0)	0.26
Coronary disease	5 (3.1)	1 (1.0)	4 (6.7)	0.13
Mental illness	1 (0.6)	1 (1.0)	0 (0.0)	0.80
Interstitial lung disease	5 (3.1)	1 (1.0)	4 (6.7)	0.13
Chronic kidney disease	5 (3.1)	1 (1.0)	4 (6.7)	0.13
Heart failure	2 (1.3)	0 (0.0)	2 (3.3)	0.27
Arterial hypertension	61 (38.1)	39 (39.0)	22 (36.7)	0.90
Obesity	6 (3.8)	4 (4.0)	2 (3.3)	0.83
Supplementary oxygen	8 (5.0)	1 (1.0)	7 (11.7)	**0.01**
OSAHS	3 (1.9)	2 (2.0)	1 (1.7)	0.65
Former/Active smoker	8 (5.0)	6 (6.0)	2 (3.3)	0.71
Tracheostomy	1 (0.6)	0 (0.0)	1 (1.7)	0.80
COPD	18 (11.3)	4 (4.0)	14 (23.3)	** < 0.01**
No conditions	62 (38.8)	43 (43.0)	19 (31.7)	0.21
Vital signs at admission, median (IQR)				
Heart rate	90.0 (78.0–103.0)	86.5 (78.0–100.3)	93.5 (78.6–110.5)	0.15
Respiratory rate	20.0 (18.0–24.3)	20.0 (18.0–25.0)	20.0 (15.0–15.0)	0.88
Glasgow score	15.0 (15.0–15.0)	15.0 (15.0–15.0)	15.0 (15.0–15.0)	**0.01**
Systolic blood pressure	120.0 (105.8–131.0)	121.0 (110.0–131.0)	116.0 (100.0–130.5)	0.20
Diastolic blood pressure	70.0 (62.8–80.0)	71.5 (65.0–80.0)	70.0 (60.0–80.0)	0,41
Treatments and interventions				
Hospital length of stay, median (IQR)	8.0 (5.0–11.0)	8.0 (5.8–12.3)	7.0 (4.0–11.0)	0.16
Mechanical ventilation, N (%)	57 (35.6)	41 (41.0)	16 (26.7)	0.10
ICU admission, N (%)	70 (43.8)	49 (49.0)	21 (35.0)	0.12
Dexamethasone, N (%)	85 (53.1)	80 (80.0)	5 (8.3)	** < 0.01**
Outcomes, N (%)				
In-hospital mortality	22 (13.8)	17 (17.0)	5 (8.3)	0.19

*IQR* Interquartile range, *OSAHS* obstructive sleep apnoea-hypopnea syndrome, *COPD* chronic obstructive pulmonary disease, *ICU* intensive care unit

### The inflammatory response in COVID-19 and CAP patients

**Fig. 1:**
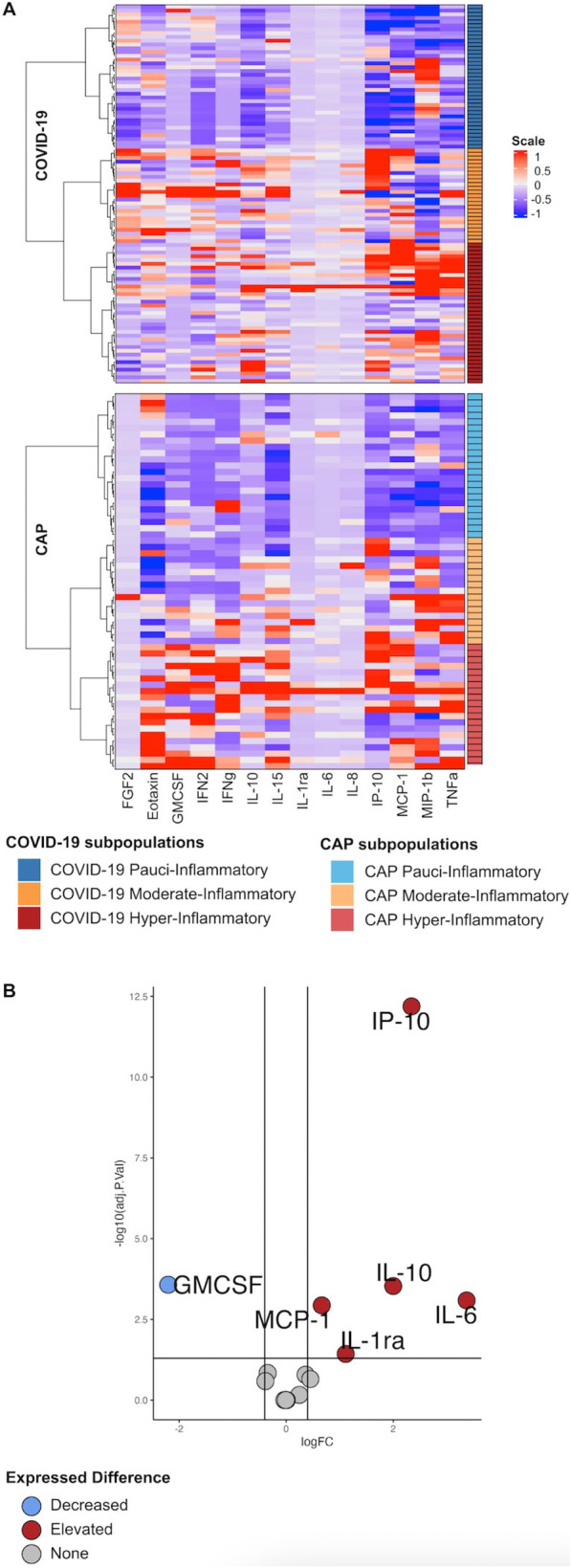
Cytokine comparison COVID-19 vs. CAP (**A**) The unsupervised heatmap model where COVID-19 and CAP patients were divided into three subpopulations using an unsupervised model, according to their cytokine levels (**B**). This volcano plot represents cytokines with a higher *P* -value and delta value in red, a higher *P*-value and lower delta value in blue, and non-significant in grey. *FGF-2* basic fibroblast growth factor, *GM-CSF* Granulocyte–macrophage colony-stimulating factor; *IFN2* Interferon α-2, *γ IFNg* interferon-g; *IL* Interleukin, *MCP-1* Monocyte chemoattractant protein-1, *MIP-1b* Macrophage inflammatory protein-1β, *TNF- α* Tumour necrosis factor-α, *CAP* Community-acquired pneumonia

We assessed the similarities and differences in inflammatory profiles, using plasma cytokines, between COVID-19 and CAP patients (Fig. 1). Compared to CAP patients, COVID-19 patients had higher IP-10 (median [IQR] 4986.8 [3044.8–8938.3] vs 749.2 [383.7–1740.8], *P*: < 0.001), IL-10 (36.2 [14.7–66.0] vs 10.6 [4.9–18.8], *P*: < 0.001), IL-6 (29.0 [9.4–60.2] vs 4.3 [0.0–47.0], *P*: 0.001), MCP-1 (562.4 [340.8–829.1] vs 321.2 [210.0–503.4], *P*: < 0.001), and IL-1α (85.7 [40.3–261.1] vs 49.7 [32.2–131.2], *P*: 0.03). In contrast, GM-CSF was lower in COVID-19 patients when compared to CAP (median [IQR] 0.7 [0.1–2.7] vs 3.8 [0.9–7.7], *P*: < 0.001).

### Three COVID-19 subpopulations were identified based on plasma cytokines (i.e., inflammatory profiles)

**Fig. 2:**
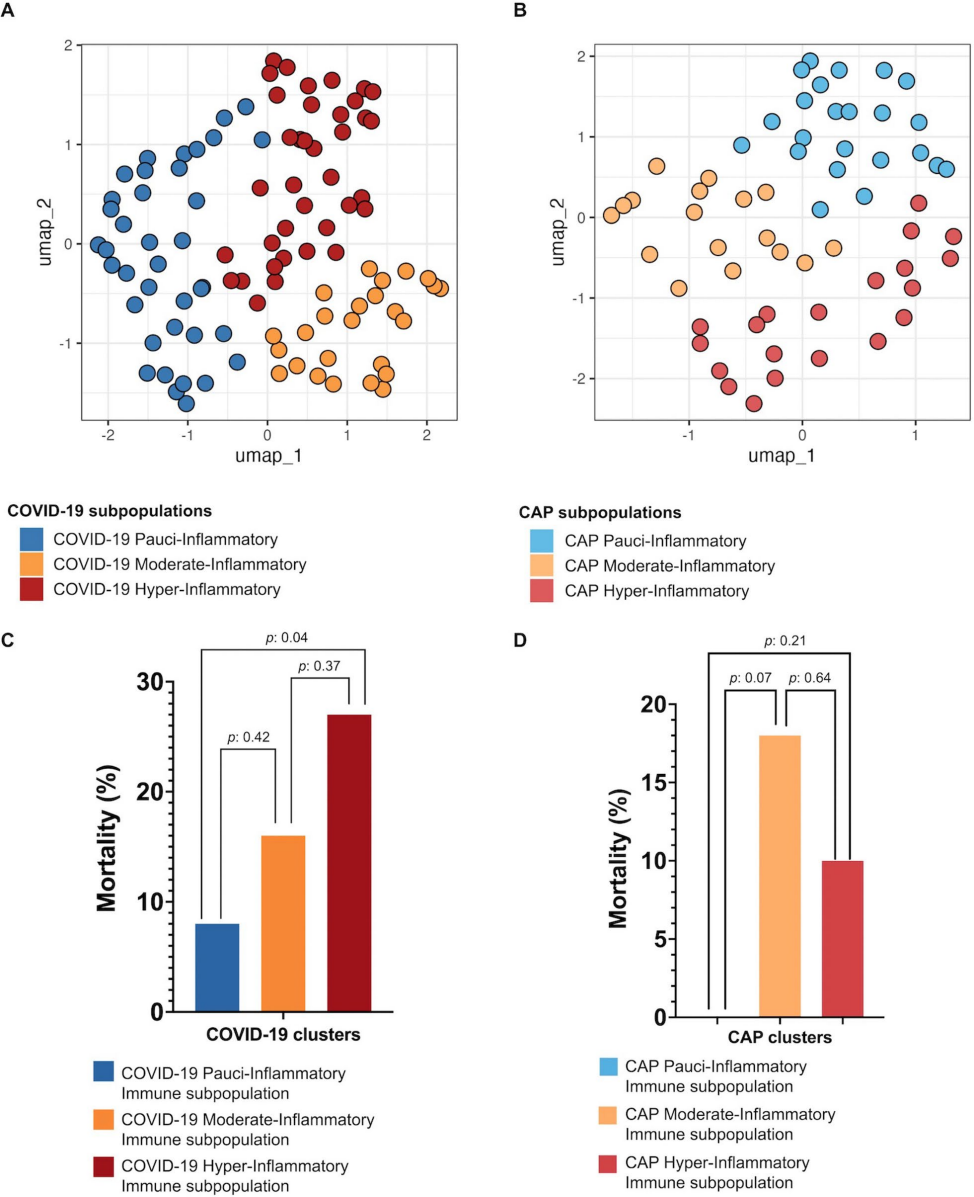
UMAP clustering and mortality comparison between COVID-19 and CAP subpopulations COVID-19 (**A**) and CAP (**B**) subpopulations are represented based on cytokine levels. COVID-19 subpopulations were compared (**C**). Only the hyper vs. pauci-inflammatory subpopulation showed a significant difference (*P* = 0.04), referring to a higher mortality rate in the hyper-inflammatory group. For CAP subpopulation comparison (**D**), no one showed a significant difference

Using an unsupervised model, COVID-19 patients were divided in three subpopulations based on its inflammatory profile: pauci-inflammatory immune response [38% (38/100)], moderate-inflammatory immune response [25% (25/100)], and hyper-inflammatory immune response [37% (37/100)]. Demographic, clinical and paraclinical data of COVID-19 subpopulations are shown in Additional file 2. Patients with hyper-inflammatory immune response had longer median [IQR] length of hospital stay (7.0 [5.0–9.8] vs 8.0 [6.0–14.0] vs 9.0 [7.0–13.0], pauci-inflammatory, moderate-inflammatory, and hyper-inflammatory; respectively), and higher hospital mortality (7.9% [3/38] vs 16.0% [4/25] vs 27.0% [10/37], pauci-inflammatory, moderate-inflammatory, and hyper-inflammatory; respectively) (Fig. 2C).

**Fig. 3:**
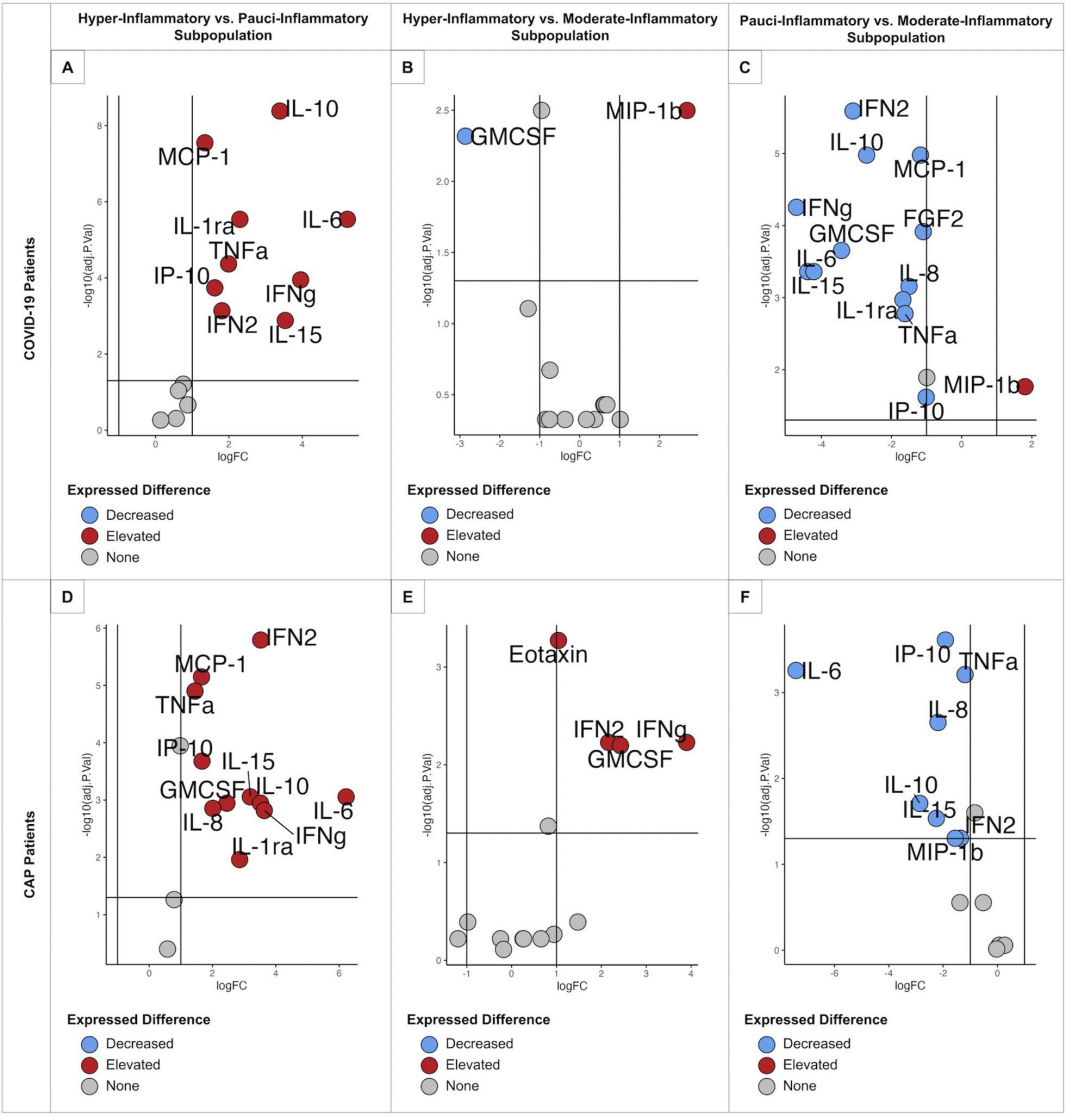
Volcano plot subpopulations This figure represents cytokines with a higher *P* -value and delta value in red, a higher *P* -value and lower delta value in blue, and non-significant in grey. COVID-19 Hyper-Inflammatory vs. Pauci-inflammatory Subpopulation (**A**), COVID-19 Hyper-Inflammatory vs. Moderate-Inflammatory Subpopulation (**B**), COVID-19 Pauci-inflammatory vs. Moderate-Inflammatory Subpopulation (**C**), CAP Hyper-Inflammatory vs. Pauci-inflammatory Subpopulation (**D**), CAP Hyper-Inflammatory vs. Moderate-Inflammatory Subpopulation (**E**), CAP Pauci-inflammatory vs. Moderate-Inflammatory Subpopulation (**F**)

Several differences in the cytokines’ concentrations were identified between subpopulations (Fig. 2A). The pauci-inflammatory cluster had lower concentrations of IP-10, MCP-1, and IL-10, while the moderate-inflammatory group had a lower concentration of MIP-1β but a higher concentration of IP-10. Finally, COVID-19 hyper-inflammatory patients had higher levels of MIP-1β, IL-10, and IP-10. Volcano plots were used to compare cytokine results in COVID-19 subpopulations. The main differences were observed when comparing hyper-inflammatory vs. pauci-inflammatory responses. As expected, hyper-inflammatory COVID-19 patients had higher concentrations of cytokines, being the most significative those from the innate immune response based on macrophages and monocytes action, such are IL-10 (median [IQR] 61.4 [42.0–109.4] vs 13.0 [5.0–24.9], *P*: < 0.001), MCP-1 (688.1 [565.9–927.1] vs 318.3 [220.3–456.6], *P*: < 0.001), IL-6 (48,1 [3–6, 6–22, 22–40] vs 9.1 [0.1–30.4], *P*: < 0.001), and IL-1α (193.3 [77.2–489.7] vs 44.2 [24.6–80.2], *P*: < 0.01) (Fig. 3).

### Three CAP subpopulations were identified based on plasma cytokines (i.e., inflammatory profiles)

CAP patients were divided in three groups based on its inflammatory profile: pauci-inflammatory [38.3% (23/60)], moderate-inflammatory [20.3% (17/60)], and hyper-inflammatory [33.3% (20/60)]. Demographic, clinical, and paraclinical data stratified by each cluster are shown in Additional file 3. Patients with moderate and hyper-inflammatory immune responses had higher mortality rates (0.0% [0/23] vs. 17.7% [3/17] vs. 10.0% [2/20], pauci-inflammatory, moderate-inflammatory, and hyper-inflammatory; respectively) (Fig. 2D).

Several differences in the cytokine’s concentrations were found between subpopulations (Fig. 2B). The moderate-inflammatory group had a higher concentration of MIP-1β, and hyper-inflammatory patients had higher concentrations of Eotaxin and IL-15. When comparing hyper-inflammatory vs. pauci-inflammatory profiles, IFN-α2 (median [IQR] 48.8 [29.7–110.5] vs 3.0 [1.7–10.3], *P*: < 0.001), MCP-1 (median [IQR] 561.3 [364.4–836.8] vs 212.6 [130.5–305.3], *P*: < 0.001), and TNF-α (median [IQR] 36.3 [24.8–53.4] vs 13.1 [11.3–16.9], *P*: < 0.001) were higher in patients with hyper-inflammatory profile (Fig. 3).

## Discussion

Clinically, patients with COVID-19 were younger, more often male, and had higher mortality than CAP patients. The dysregulated inflammatory responses measured using plasma cytokine concentrations appear different between COVID-19 and CAP, with COVID-19 patients showing higher levels of IP-10, IL-10, and IL-6, and CAP patients had higher levels of GM-CSF. Although we identified three sub-populations with unsupervised clustering in COVID-19 and CAP, the inflammatory profiles differed between subpopulations, indicating biological heterogeneity and biological differences between COVID-19 and CAP that could inform future clinical trials in these two conditions.

The clinical presentation of COVID-19 and CAP are very similar. Both can vary from mild respiratory symptoms and progress to sepsis and respiratory failure. Also, risk factors for developing severe infection include chronic cardiovascular disease, respiratory diseases, and diabetes, which correlate with our findings [13, 14]. Our study found that most patients with COVID-19 have mild or moderate disease, but up to 30% of patients with COVID-19 require advanced ventilatory support, similar to previous reports [15, 16]. It has been documented during the pandemic that 10–20% of COVID-19 patients require ICU admission, of which 40–80% need mechanical ventilation [17], with a mortality rate that can vary from 5 to 36%, depending on disease severity [18, 19]. In our study, COVID-19 patients had higher ICU requirement, mechanical ventilation, and mortality rate when compared to CAP patients, which is a novel finding. These differences could be attributed to the fact that COVID-19 and CAP have underlying immune mechanisms [20]. For instance, immunomodulatory treatments with corticosteroids or interleukin modulators are now the standard of care for patients with COVID-19, but not for patients with CAP [21–25]. For example, in CAP patients, the current thinking is that patients with documented higher inflammation, confirmed by high serum C reactive protein (CRP), may benefit from treatment with steroids [26], and the dosing of corticosteroids is different between the two conditions [27].

The main differences between COVID-19 and CAP lie in their immunopathology. Severe COVID-19 inflammatory response is characterized by a hyperinflammatory phase in some patients that could drive a metabolic reprogramming of immune cells, such as neutrophils [28, 29] and generate intra-pulmonary inflammatory circuits [30]. The cytokine profile we report in our cohort and those associated hyper-inflammatory subpopulations are consistent with the published literature [31–33]. Leisman D et al. showed in a metanalysis that even though the concentration of these cytokines in critically ill COVID-19 patients is high, it does not exceed other inflammatory disorders such as chimeric antigen receptor (CAR) T cell-induced cytokine release syndrome, sepsis, and unrelated COVID-19 associated acute respiratory distress syndrome (ARDS) [34]. In sharp contrast, CAP immune reaction depends on its etiological pathogen. For instance, *Streptococcus pneumoniae* can generate local and systemic inflammation by activating pro-inflammatory cell death pathways (i.e., necroptosis and pyroptosis) in alveolar epithelial cells and alveolar macrophages [35]. Also, pneumolysin, a pore-forming toxin produced by *S. pneumoniae*, can generate severe inflammation by directly activating the NLRP3 inflammasome through IL-1β and IL-17A [36]. These differences support our results, where innate immune responses among COVID-19 and CAP are different. Therefore, we hypothesize that when evaluating patients with COVID-19 and CAP, using serum biomarkers could inform immunomodulation treatment choices and identify subpopulations of interest [37].

Cytokine concentrations and ratios may predict the development of severe infection in COVID-19 patients [29]. In favour of this argument, McElvaney O et al. showed in a comprehensive series of experiments that the metabolic reprogramming activation led by cytokines in severe COVID-19 patients causes profound change to their function, ending in organ failure ICU need [29]. Later in the pandemic, we found that circulating autoantibodies neutralizing high concentrations of IFN-α and/or IFN-ω are found in about 10% of patients with critical COVID-19 pneumonia but not in individuals with asymptomatic infections, representing about 20% of both critical COVID-19 cases fatal COVID-19 cases [38]. Finally, Martinez-Fleta P et al*.* attempted to find differences in the mechanisms of host–pathogen interaction among COVID-19 and CAP patients. They found a differential profile in circulating miRNAs between COVID-19 and CAP patients, indicating that SARS-CoV-2 can induce more systemic tissue damage [39, 40]. Punctually, they found that COVID-19 cytokine dysregulation recruits lymphocytes, leading to systemic and pulmonary injury, resulting in organ failure related to higher mortality among hyper-inflammatory COVID-19 patients. Our results, along with the work described before, build into the argument that COVID-19 and CAP have different infectious mechanisms that require different treatments. Our results highlight the importance of developing reliable near-patient biomarker measuring devices to identify subpopulations in COVID-19 and CAP patients to enable precision therapeutics.

Our study has some limitations and strengths that are important to acknowledge. First, this is a monocentric study that limits the results generalizability. However, we focused on understanding the underlying mechanisms of the host–pathogen interactions in COVID-19 and CAP, which should be similar in all patients admitted to hospitals globally. Second, the number of patients enrolled in the study was small. Notably, we performed a comprehensive molecular approach, including unsupervised analyses. Third, we do not have a healthy group as control subjects, but our goal was to compare COVID-19 and CAP immune profiles. These differences between COVID-19 and CAP patients support our hypothesis that the underlying immune mechanisms following the host–pathogen interactions during these conditions differ. Importantly, we did not identify the mechanisms for these differences, including sub-populations, which should be addressed in future studies. Finally, we did not collect data about the number of days with symptoms before the hospital admission, which might be considered a limitation. However, we use data at hospital admission, which is a more homogeneous time-point to compare patients.

## Conclusion

We report similarities and differences in cytokine profiles from patients with COVID-19 and CAP. Also, we identified three sub-populations in both diseases based on their inflammatory profiles. Moreover, we found that these inflammatory profiles are associated with different clinical outcomes. Thus, each patient should be assessed based on their clinical and paraclinical profiles to identify the best treatment, determine disease severity and predict the clinical outcomes. Finally, these results support that inflammation in patients with COVID-19 and CAP is different; thus, therapeutic targets should be individualized for both diseases.

## Supplementary Information

**Additional file 1: Fig S1.** Marker enrichment modelling (MEM). This model was used to identify discriminant cytokines of each group, (**A**) correspond to COVID-19 patients and (**B**) to CAP patients.

**Additional file 2: Table S1. **Baseline characteristics of COVID-19 subpopulations.

**Additional file 3: Table S2. **Baseline characteristics of CAP subpopulations.

## Data Availability

The datasets used and/or analysed during the current study are available from the corresponding author on reasonable request.
