# Supplementary material for: Comparison of systemic inflammatory profiles in COVID-19 and community-acquired pneumonia patients: a prospective cohort study
Source: Respir Res. 2023 Feb 22;24:60. doi: 10.1186/s12931-023-02352-2 (PMC9944840; doi:10.1186/s12931-023-02352-2)
Supplement: Supplementary file 1 — Additional file 1: Fig S1. Marker enrichment modelling (MEM). This model was used to identify discriminant cytokines of each group, (A) correspond to COVID-19 patients and (B) to CAP patients. [file 12931_2023_2352_MOESM1_ESM.docx]

**TITLE:** Comparison of Systemic Inflammatory Profiles in COVID-19 and Community-Acquired Pneumonia Patients: A Prospective Cohort Study.

**AUTHORS:** Elsa D. Ibáñez-Prada^1#^, Matthew Fish,^2#^ Yuli V. Fuentes,^1,3^, Ingrid G. Bustos,^1#^ Cristian C. Serrano-Mayorga,^1,3^ Julian Lozada,^1^ Jennifer Rynne,^2^ Aislinn Jennings,^2^ Ana M. Crispin,^3^ Ana Maria Santos,^1^ John Londoño,^1^ Manu Shankar-Hari^2##*^ and Luis Felipe Reyes^1,3,4 ##*^.

#Co-first authors.

##Co-corresponding authors.

**AFFILIATIONS:** 1, Universidad de La Sabana, Chia, Colombia; 2, Centre for Inflammation Research, University of Edinburgh; 47 Little France Crescent, Edinburgh, Scotland; United Kingdom; 3, Clínica Universidad de La Sabana, Chía, Colombia; 4, Nuffield School of Medicine, University of Oxford, Oxford, United Kingdom.

**Author for Correspondence:** Luis Felipe Reyes, MD, PhD; Universidad de La Sabana, Campus Puente del Común, KM 7.5 Autopista Norte de Bogotá, Chía, Colombia. Phone: (571)-861-5555 ext. 23342; Email: [luis.reyes5@unisabana.edu.co](mailto:luis.reyes5@unisabana.edu.co)

**Additional file 1:** **Fig S1. Marker enrichment modelling (MEM)** This model was used to identify discriminant cytokines of each group, (A) correspond to COVID-19 patients and (B) to CAP patients.

**
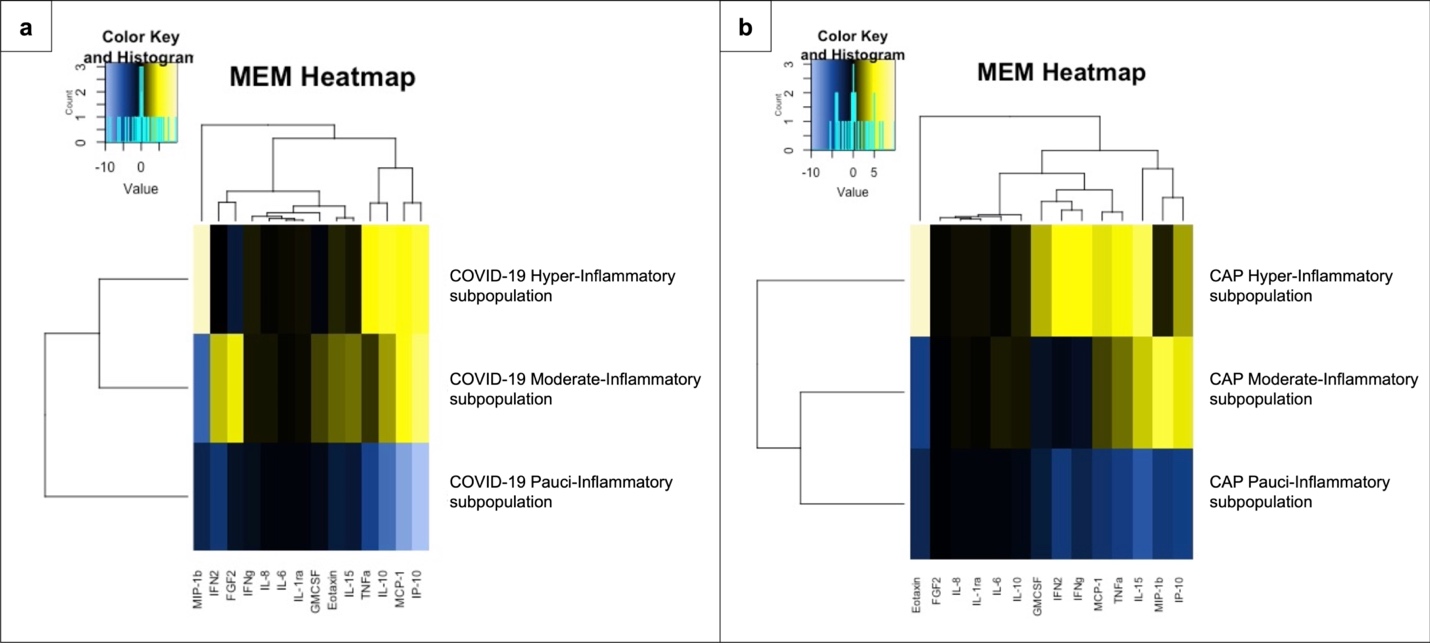
**
