# Supplementary material for: Comparison of systemic inflammatory profiles in COVID-19 and community-acquired pneumonia patients: a prospective cohort study
Source: Respir Res. 2023 Feb 22;24:60. doi: 10.1186/s12931-023-02352-2 (PMC9944840; doi:10.1186/s12931-023-02352-2)
Supplement: Supplementary file 2 — Additional file 2: Table S1. Baseline characteristics of COVID-19 subpopulations. [file 12931_2023_2352_MOESM2_ESM.docx]

**TITLE:** Comparison of Systemic Inflammatory Profiles in COVID-19 and Community-Acquired Pneumonia Patients: A Prospective Cohort Study.

**AUTHORS:** Elsa D. Ibáñez-Prada^1#^, Matthew Fish,^2#^ Yuli V. Fuentes,^1,3^, Ingrid G. Bustos,^1#^ Cristian C. Serrano-Mayorga,^1,3^ Julian Lozada,^1^ Jennifer Rynne,^2^ Aislinn Jennings,^2^ Ana M. Crispin,^3^ Ana Maria Santos,^1^ John Londoño,^1^ Manu Shankar-Hari^2##*^ and Luis Felipe Reyes^1,3,4 ##*^.

#Co-first authors.

##Co-corresponding authors.

**AFFILIATIONS:** 1, Universidad de La Sabana, Chia, Colombia; 2, Centre for Inflammation Research, University of Edinburgh; 47 Little France Crescent, Edinburgh, Scotland; United Kingdom; 3, Clínica Universidad de La Sabana, Chía, Colombia; 4, Nuffield School of Medicine, University of Oxford, Oxford, United Kingdom.

**Author for Correspondence:** Luis Felipe Reyes, MD, PhD; Universidad de La Sabana, Campus Puente del Común, KM 7.5 Autopista Norte de Bogotá, Chía, Colombia. Phone: (571)-861-5555 ext. 23342; Email: [luis.reyes5@unisabana.edu.co](mailto:luis.reyes5@unisabana.edu.co)

**Additional file 2:** **Table S1.** Baseline characteristics of COVID-19 subpopulations.

| **Characteristic** | **COVID-19 pauci-inflammatory immune response**  **(N= 38)** | **COVID-19 moderate-inflammatory immune response**  **(N= 25)** | **COVID-19 hyper-inflammatory immune response**  **(N= 37)** | ***P*-value** |
| --- | --- | --- | --- | --- |
| Male gender, N (%) | 22 (57.9) | 26 (70.3) | 17 (68.0) | 0.50 |
| Age, median (IQR) | 54.5 (42.5 – 63.0) | 59.0 (54.0 – 68.0) | 58.0 (51.0 – 67.0) | 0.16 |
| **Comorbid conditions, N (%)** | | | | |
| Stroke | 0 (0.0) | 1 (4.0) | 0 (0.0) | 0.22 |
| Myocardial infarction | 0 (0.0) | 0 (0.0) | 0 (0.0) | 1.00 |
| Heart arrhythmia | 0 (0.0) | 1 (4.0) | 1 (2.7) | 0.50 |
| Asthma | 0 (0.0) | 0 (0.0) | 0 (0.0) | 1.00 |
| Bronchiectasis | 0 (0.0) | 0 (0.0) | 0 (0.0) | 1.00 |
| Active cancer | 0 (0.0) | 0 (0.0) | 1 (2.7) | 0.42 |
| Dementia | 0 (0.0) | 0 (0.0) | 0 (0.0) | 1.00 |
| Diabetes mellitus | 2 (5.3) | 1 (4.0) | 5 (13.5) | 0.29 |
| Coronary disease | 0 (0.0) | 1 (4.0) | 0 (0.0) | 0.22 |
| Mental illness | 0 (0.0) | 0 (0.0) | 1 (2.7) | 0.42 |
| Intersticial lung disease | 1 (2.6) | 0 (0.0) | 0 (0.0) | 0.44 |
| Chronic kidney disease | 0 (0.0) | 0 (0.0) | 1 (2.7) | 0.42 |
| Heart failure | 0 (0.0) | 0 (0.0) | 0 (0.0) | 1.00 |
| Arterial hypertension | 14 (36.8) | 10 (40.0) | 15 (40.5) | 0.94 |
| Obesity | 3 (7.9) | 0 (0) | 1 (2.7) | 0.26 |
| Supplementary oxygen | 0 (0.0) | 1 (4.0) | 0 (0.0) | 0.22 |
| OSAHS | 1 (2.6) | 0 (0) | 1 (2.7) | 0.71 |
| Former/Active smoker | 2 (5.3) | 2 (8.0) | 2 (5.4) | 0.89 |
| Tracheostomy | 0 (0.0) | 0 (0.0) | 0 (0.0) | 1.00 |
| COPD | 2 (5.3) | 1 (4.0) | 1 (2.7) | 0.85 |
| No conditions | 20 (52.6) | 11 (44.0) | 12 (32.4) | 0.21 |
| **Vital signs at admission, median (IQR)** | | | | |
| Heart rate | 85.5 (75.3 – 97.3) | 86.0 (80.0 – 98.0) | 90.0 (80.0 – 104.0) | 0.31 |
| Respiratory rate | 20.0 (18.0 – 25.8) | 21.0 (18.0 – 25.0) | 20.0 (18.0 – 24.0) | 0.68 |
| Glasgow score | 15.0 (15.0 – 15.0) | 15.0 (15.0 – 15.0) | 15.0 (15.0 – 15.0) | 0.22 |
| Systolic blood pressure | 122.0 (110.3 – 130.8) | 126.0 (110.0 – 140.0) | 120.0 (110.0 – 130.0) | 0.49 |
| Diastolic blood pressure | 71.5 (66.0 – 80.0) | 72.0 (67.0 – 80.0) | 70.0 (65.0 – 80.0) | 0.79 |
| **Treatments and interventions** | | | | |
| Hospital length of stay, median (IQR) | 7.0 (5.0 – 9.8) | 8.0 (6.0 – 14.0) | 9.0 (7.0 – 13.0) | 0.32 |
| Mechanical ventilation, N (%) | 15 (39.5) | 9 (36.0) | 17 (46.0) | 0.72 |
| ICU admission, N (%) | 17 (44.7) | 13 (52.0) | 19 (51.4) | 0.80 |
| Dexamethasone, N (%) | 29 (76.3) | 20 (80.0) | 31 (83.8) | 0.72 |
| **Outcomes, N (%)** | | | | |
| In-hospital mortality | 3 (7.9) | 4 (16.0) | 10 (27.0) | 0.09 |

Abbreviation: IQR: Interquartile range; OSAHS: obstructive sleep apnea-hypopnea syndrome; COPD: chronic obstructive pulmonary disease; ICU: intensive care unit.
