# Supplementary material for: Comparison of systemic inflammatory profiles in COVID-19 and community-acquired pneumonia patients: a prospective cohort study
Source: Respir Res. 2023 Feb 22;24:60. doi: 10.1186/s12931-023-02352-2 (PMC9944840; doi:10.1186/s12931-023-02352-2)
Supplement: Supplementary file 3 — Additional file 3: Table S2. Baseline characteristics of CAP subpopulations. [file 12931_2023_2352_MOESM3_ESM.docx]

**TITLE:** Comparison of Systemic Inflammatory Profiles in COVID-19 and Community-Acquired Pneumonia Patients: A Prospective Cohort Study.

**AUTHORS:** Elsa D. Ibáñez-Prada^1#^, Matthew Fish,^2#^ Yuli V. Fuentes,^1,3^, Ingrid G. Bustos,^1#^ Cristian C. Serrano-Mayorga,^1,3^ Julian Lozada,^1^ Jennifer Rynne,^2^ Aislinn Jennings,^2^ Ana M. Crispin,^3^ Ana Maria Santos,^1^ John Londoño,^1^ Manu Shankar-Hari^2##*^ and Luis Felipe Reyes^1,3,4 ##*^.

#Co-first authors.

##Co-corresponding authors.

**AFFILIATIONS:** 1, Universidad de La Sabana, Chia, Colombia; 2, Centre for Inflammation Research, University of Edinburgh; 47 Little France Crescent, Edinburgh, Scotland; United Kingdom; 3, Clínica Universidad de La Sabana, Chía, Colombia; 4, Nuffield School of Medicine, University of Oxford, Oxford, United Kingdom.

**Author for Correspondence:** Luis Felipe Reyes, MD, PhD; Universidad de La Sabana, Campus Puente del Común, KM 7.5 Autopista Norte de Bogotá, Chía, Colombia. Phone: (571)-861-5555 ext. 23342; Email: [luis.reyes5@unisabana.edu.co](mailto:luis.reyes5@unisabana.edu.co)

**Additional file 3: Table S2.** Baseline characteristics of CAP subpopulations.

| **Characteristic** | **CAP pauci-inflammatory immune response**  **(N= 23)** | **CAP moderate-inflammatory immune response**  **(N= 17)** | **CAP hyper-inflammatory immune response**  **(N= 20)** | ***P*-value** |
| --- | --- | --- | --- | --- |
| Male gender, N (%) | 13 (56.5) | 11 (64.7) | 8 (40.0) | 0.30 |
| Age, median (IQR) | 63.0 (51.5 – 78.5) | 65.0 (52.0 – 78.0) | 66.5 (50.0 – 77.0) | 0.77 |
| **Comorbid conditions, N (%)** | | | | |
| Stroke | 0 (0.0) | 1 (5.9) | 3 (15.0) | 0.14 |
| Myocardial infarction | 2 (8.7) | 1 (5.9) | 0 (0.0) | 0.42 |
| Heart arrhythmia | 0 (0.0) | 1 (5.9) | 1 (5.0) | 0.52 |
| Asthma | 2 (8.7) | 0 (0.0) | 0 (0.0) | 1.19 |
| Bronchiectasis | 1 (4.4) | 0 (0.0) | 0 (0.0) | 0.44 |
| Active cancer | 1 (4.4) | 1 (5.9) | 1 (5.0) | 0.98 |
| Dementia | 1 (4.4) | 0 (0.0) | 1 (5.0) | 0.66 |
| Diabetes mellitus | 2 (8.7) | 3 (17.7) | 4 (20.0) | 0.55 |
| Coronary disease | 0 (0.0) | 1 (5.9) | 3 (15.0) | 0.14 |
| Mental illness | 0 (0.0) | 0 (0.0) | 0 (0.0) | 1.00 |
| Intersticial lung disease | 2 (8.7) | 1 (5.9) | 1 (5.0) | 0.88 |
| Chronic kidney disease | 2 (8.7) | 1 (5.9) | 1 (5.0) | 0.88 |
| Heart failure | 1 (4.4) | 0 (0.0) | 1 (5.0) | 0.66 |
| Arterial hypertension | 9 (39.1) | 6 (35.3) | 7 (35.0) | 0.95 |
| Obesity | 2 (8.7) | 0 (0.0) | 0 (0.0) | 0.19 |
| Supplementary oxygen | 3 (13.0) | 0 (0.0) | 4 (20.0) | 0.16 |
| OSAHS | 0 (0.0) | 0 (0).0 | 1 (5.0) | 0.36 |
| Former/Active smoker | 1 (4.4) | 1 (5.9) | 0 (0.0) | 0.58 |
| Tracheostomy | 1 (4.4) | 0 (0.0) | 0 (0.0) | 0.44 |
| COPD | 6 (26.1) | 3 (17.7) | 5 (25.0) | 0.80 |
| No conditions | 6 (26.1) | 6 (35.3) | 7 (35.0) | 0.76 |
| **Vital signs at admission, median (IQR)** | | | | |
| Heart rate | 86.0 (78.0 – 104.0) | 92.0 (79.0 – 100.0) | 101.5 (83.8 – 116.3) | 0.29 |
| Respiratory rate | 22.0 (19.5 – 23.0) | 20.0 (18.0 – 22.0) | 20.0 (18.0 – 25.0) | 0.65 |
| Glasgow score | 15.0 (15.0 – 15.0) | 15.0 (15.0 – 15.0) | 15.0 (15.0 – 15.0) | 0.19 |
| Systolic blood pressure | 128.0 (109.5 – 141.5) | 116.0 (110.0 – 130.0) | 110.0 (96.0 – 120.5) | 0.05 |
| Diastolic blood pressure | 71.0 (63.5 – 80.0) | 66.0 (60.0 – 79.0) | 70.0 (60.0 – 80.0) | 0.43 |
| **Treatments and interventions** | | | | |
| Hospital length of stay, median (IQR) | 7.0 (3.5 – 9.5) | 8.0 (5.0 – 14.0) | 6.5 (4.0 – 9.3) | 0.43 |
| Mechanical ventilation, N (%) | 5 (21.7) | 6 (35.3) | 5 (25.0) | 0.62 |
| ICU admission, N (%) | 6 (26.1) | 7 (41.2) | 8 (40.0) | 0.52 |
| Dexamethasone, N (%) | 2 (8.7) | 3 (17.7) | 0 (0.0) | 0.15 |
| **Outcomes, N (%)** | | | | |
| In-hospital mortality | 0 (0.0) | 3 (17.7) | 2 (10.0) | 0.13 |

Abbreviation: IQR: Interquartile range; OSAHS: obstructive sleep apnea-hypopnea syndrome; COPD: chronic obstructive pulmonary disease; ICU: intensive care unit.
